# Self-managed, computerised word finding therapy as an add-on to usual care for chronic aphasia post-stroke: An economic evaluation

**DOI:** 10.1177/0269215520975348

**Published:** 2020-11-24

**Authors:** Nicholas R Latimer, Arjun Bhadhuri, Abualbishr Alshreef, Rebecca Palmer, Elizabeth Cross, Munyaradzi Dimairo, Steven Julious, Cindy Cooper, Pam Enderby, Marian C Brady, Audrey Bowen, Ellen Bradley, Madeleine Harrison

**Affiliations:** 1 ScHARR, The University of Sheffield, Sheffield, UK; 2 Sheffield Clinical Trials Research Unit, The University of Sheffield, Sheffield, UK; 3 NMAHP Research Unit, Glasgow Caledonian University, Glasgow, UK; 4 Division of Neuroscience & Experimental Psychology, FBMH, University of Manchester, MAHSC, Manchester, UK

**Keywords:** Aphasia, speech therapy, cost-effectiveness, computer supported, self-management, Cost-Effectiveness, Health Services, Rehabilitation, Treatment

## Abstract

**Objective:**

To examine the cost-effectiveness of self-managed computerised word finding therapy as an add-on to usual care for people with aphasia post-stroke.

**Design:**

Cost-effectiveness modelling over a life-time period, taking a UK National Health Service (NHS) and personal social service perspective.

**Setting:**

Based on the Big CACTUS randomised controlled trial, conducted in 21 UK NHS speech and language therapy departments.

**Participants:**

Big CACTUS included 278 people with long-standing aphasia post-stroke.

**Interventions:**

Computerised word finding therapy plus usual care; usual care alone; usual care plus attention control.

**Main measures:**

Incremental cost-effectiveness ratios (ICER) were calculated, comparing the cost per quality adjusted life year (QALY) gained for each intervention. Credible intervals (CrI) for costs and QALYs, and probabilities of cost-effectiveness, were obtained using probabilistic sensitivity analysis. Subgroup and scenario analyses investigated cost-effectiveness in different subsets of the population, and the sensitivity of results to key model inputs.

**Results:**

Adding computerised word finding therapy to usual care had an ICER of £42,686 per QALY gained compared with usual care alone (incremental QALY gain: 0.02 per patient (95% CrI: −0.05 to 0.10); incremental costs: £732.73 per patient (95% CrI: £674.23 to £798.05)). ICERs for subgroups with mild or moderate word finding difficulties were £22,371 and £21,262 per QALY gained respectively.

**Conclusion:**

Computerised word finding therapy represents a low cost add-on to usual care, but QALY gains and estimates of cost-effectiveness are uncertain. Computerised therapy is more likely to be cost-effective for people with mild or moderate, as opposed to severe, word finding difficulties.

## Introduction

Aphasia is a language disorder which causes problems with reading, writing, talking and/or understanding spoken language.^1^ Aphasia can restrict participation in work, family and community life. Approximately 33% of people who have a stroke experience aphasia, and 30%–43% of these remain significantly affected in the long-term.^2^ However, people with aphasia can improve with speech and language therapy,^3^ and acceptability and demand for ongoing therapy is high,^4^ but availability can be limited around the world due to staffing and budgetary constraints.^5–7^

A computerised approach to word finding therapy (hereafter referred to as computerised therapy) has the potential to increase access to speech and language therapy, because it enables patients to self-manage repetitive language exercises without the presence of a speech and language therapist. This may be particularly helpful in health systems with constrained resources. Many health systems use economic evaluation to ensure that limited healthcare budgets are allocated efficiently.

Big CACTUS (Cost effectiveness of Aphasia Computer Therapy versus Usual care or attention control post Stroke) represented the first multicentre randomised controlled trial (RCT) investigating computer therapy for word finding in aphasia.^8^ The trial built on an earlier pilot trial, named CACTUS.^9^ An economic evaluation undertaken alongside the CACTUS pilot trial indicated that computerised therapy may represent a cost-effective use of healthcare resources, but was highly uncertain due to the small sample size – it was concluded that further research was necessary.^10^ Big CACTUS found that adding computerised therapy to usual care statistically and clinically significantly improved word finding ability but the effect did not generalise to measures of conversation.^11,12^ Improved ability to find words represents an important step towards improved communication and therefore – despite the need for further research related to methods of generalising the effect to conversation – we sought to use evidence from Big CACTUS to undertake an updated economic evaluation investigating the long-term cost-effectiveness of adding computerised therapy to usual care for people with aphasia post-stroke.

## Methods

### Big CACTUS

Big CACTUS was a pragmatic, superiority, observer-blinded, parallel group, RCT conducted in 21 UK NHS speech and language therapy departments. Participants were recruited between Oct 20, 2014 and Aug 18, 2016, and were followed up between Oct 24, 2015 and Sept 12, 2017. The trial was registered with the ISRCTN registry [number ISRCTN68798818]. Ethics approval was obtained from Leeds West NHS research ethics committee [reference 13/YH/0377] and Scotland A research ethics committee [reference 14/SS/0023] and written informed consent was obtained from participants or their carers.^11,12^ The trial was funded by the National Institute for Health Research (NIHR) [reference 12/21/01] and the Tavistock Trust for Aphasia. The trial protocol is publicly available^8^ and specific details on clinical aspects of the study can be found in an extensive report for the funder (which also includes full details of the economic evaluation),^11^ and have been summarised elsewhere.^12^ The CACTUS pilot study was also registered with the ISRCTN registry [number ISRCTN91534629], was funded by the NIHR [reference PB-PG-1207-14097], and clinical and cost-effectiveness results are published.^9,13^

In Big CACTUS, participants were randomised into three groups: (1) computerised word finding therapy plus usual care, (2) attention control plus usual care and (3) usual care alone. Computerised therapy involved aphasia therapy software (StepByStep^©^) tailored to the participant’s language impairment needs and personalised with 100 words relevant to the participant by a speech and language therapist. The participant was encouraged to practise word finding for six months on a daily basis. The intervention included monthly support from a speech and language therapist assistant or volunteer. The attention control group received puzzle books and monthly supportive telephone calls. Usual care (including speech and language therapy) continued to be provided to patients in all intervention groups, so that the effectiveness of computerised therapy as an addition to usual care could be assessed, rather than investigating computerised therapy as a replacement for usual care.

Participants had aphasia confirmed by a speech and language therapist after one or more strokes at least four months before randomisation – though many experienced their stroke much longer ago; the median time post stroke was approximately two years. Participants had word finding difficulties (defined by a score of 5–43/48 on the Comprehensive Aphasia Test (CAT) Naming Objects test^14^), could perform a simple matching task on StepByStep^©^ software with at least 50% accuracy, and could repeat at least 50% of words in a repetition task on StepByStep^©^. The average age of participants was 65 years.

### Economic evaluation of computerised therapy: Overview

A model-based cost-utility analysis was conducted in line with recommendations made by the National Institute for Health and Care Excellence (NICE), the UK health technology assessment agency,^15^ taking a UK NHS and personal social services perspective (where personal social services refer to services provided by local authorities which are funded by the NHS). Due to the potentially long-lasting effects of the intervention a lifetime horizon was modelled, meaning that the patient experience was modelled until all patients were projected to have died. The incremental costs and benefits of the treatment arms evaluated in Big CACTUS were assessed, and the population modelled was that included in Big CACTUS. Therefore clinical effectiveness estimates (and distributions around these estimates) were taken from the clinical measures used in the trial.^11^

In line with NICE recommendations,^15^ benefits were calculated in terms of quality adjusted life years (QALYs) which combine length of life and health-related quality of life into one measure. The costs and QALYs associated with each treatment option were compared and combined into incremental cost-effectiveness ratios (ICERs). ICERs express as a ratio the incremental costs of a new intervention relative to the incremental QALYs it produces: therefore, we estimated the incremental cost of computerised therapy plus usual care compared to usual care alone, and estimated the incremental QALY gain associated with computerised therapy plus usual care compared to usual care alone, and distilled these estimates into a ratio: the incremental cost per QALY gained, also known as the ICER. We did the same for the comparison of computerised therapy plus usual care compared to attention control plus usual care, and therefore obtained ICERs for computerised therapy plus usual care compared to usual care alone, and compared to attention control plus usual care. ICERs were compared to thresholds used by NICE to aid decision making. NICE typically considers interventions to be cost-effective if the ICER is less than £20,000–£30,000 per QALY gained ($26,023–$39,035 in United States Dollars, at an exchange rate of £1:$1.30).^15^

The economic model incorporated several assumptions and model inputs (e.g. around treatment effectiveness, costs and quality of life), to be described in subsequent sections. We conducted a ‘base-case’ analysis which incorporated our preferred assumptions for each model input and modelled the full patient population included in Big CACTUS. However, recognising that some model inputs were particularly uncertain, we conducted a series of pre-specified secondary analyses, specifically around different cost assumptions and different methods for estimating health-related quality of life scores. We also conducted pre-specified subgroup analyses for subsets of the Big CACTUS population. Subgroup analyses for word finding difficulty at baseline (mild/moderate/severe, identified using scores from the CAT Naming Objects test at baseline) are presented here. Other subgroup analyses (Comprehension ability subgroups; Time since stroke subgroups) are reported elsewhere.^11^

### Model structure

**Figure 1.**
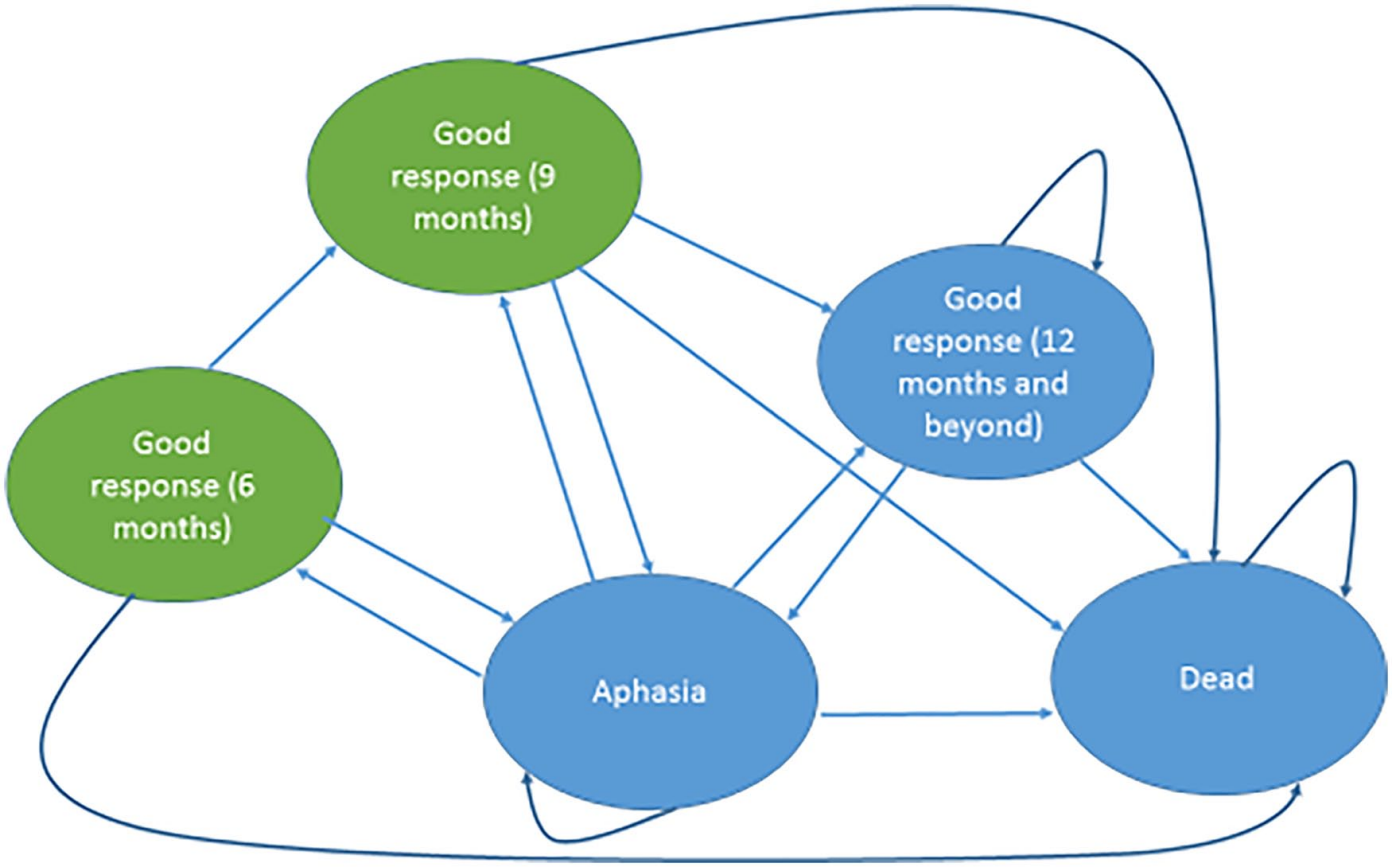
Markov model structure. Each oval represents a health state. Participants begin in the ‘Aphasia’ health state and transition through the model in three-month cycles according to data on response and relapse from Big CACTUS. Arrows illustrate possible pathways through the model. Health states coloured in green represent ‘tunnel states’, which means that participants can only reside in these states for one modelled cycle before transitioning to a different health state. Death could occur from any health state. No new responses were assumed to occur after 12 months – from that point onwards participants in the ‘Good response (12 months and beyond)’ health state either retain a good response, relapse to the ‘Aphasia’ health state or die. From 12 months onwards people in the ‘Aphasia’ health state either remain in that health state or die.

A Markov model was used. This is a commonly used type of economic model that consists of a series of ‘health states’, representing disease status.^16^ People move between the health states over time, allowing key changes in disease status to be modelled (Figure 1). The model begins with all people in the ‘Aphasia’ health state. Every three months, people were modelled to move between the different health states in the model (‘Aphasia’; ‘Good response’; ‘Dead’), or remain in their existing health state (i.e. a three month cycle length was used). Death could occur from any health state. For the first 12 months of the model, people transitioned through the model according to clinical effectiveness data from Big CACTUS, which collected outcomes data at six, nine and 12 month time-points. For instance, a participant who, according to Big CACTUS data, had a good response at six months, would be placed in the ‘Good response (six months)’ health state at the six-month time-point in the model. The ‘Good response (six months)’ health state is a ‘tunnel’ state, which means that people could only spend three months (one modelled cycle) in it. At the following modelled time point (nine months, given the three-month cycle length used), the participant could either maintain their good response and move into the ‘Good response (nine months)’ health state, could relapse to the ‘Aphasia’ health state or could die. The ‘Good response (nine months)’ health state is also a ‘tunnel’ state, so people could only spend three months (one modelled cycle) in it. So, a participant with a good response at nine months could either retain that response and move into the ‘Good response (12 months and beyond)’ health state in the following modelled cycle, could relapse to the ‘Aphasia’ health state, or could die. Big CACTUS did not measure outcomes after 12 months, and so we assumed no new responses beyond this time-point. Hence, a patient who had not achieved a good response at the 12 month time-point would remain in the ‘Aphasia’ health state until death. A patient with a good response measured at 12 months could subsequently maintain that response and remain in the ‘Good response (12 months and beyond)’ health state, could relapse to the ‘Aphasia’ health state, or could die.

### Model parameters

A good response was characterised as a clinically meaningful improvement (from baseline) in either of the co-primary outcome measures included in Big CACTUS, pre-specified as an increase of 10% or more in words found correctly on a naming test of 100 personally relevant words, and/or an increase of 0.5 points or more on the Therapy Outcomes Measures activity scale.^17^ This was pre-specified in the publicly available health economics analysis plan,^17^ as it was considered that any patient who achieved either of these improvements could be considered to have responded well to treatment. Model transitions from the ‘Good response’ health states back to the ‘Aphasia’ health state were determined by an analysis of the changing response proportions over time observed in each treatment arm of Big CACTUS.

Beyond 12 months (the final data collection point in Big CACTUS), we assumed that no new good responses occurred in any treatment arm. The relapse rate observed between nine and 12 months was assumed to remain constant for the remainder of the modelled period, hence we assumed that good responses were lost over time.

A proportion of participants were assumed to die in each model cycle, based on post-stroke death rates combined with age-related mortality risks.^18,19^ Mortality rates were the same in each health state of the model and for each intervention – therefore it was assumed that the interventions under consideration did not affect mortality or life expectancy. Mortality rates in the economic model simply reflect expected death rates post-stroke over time.

### Health related quality of life

NICE recommends the EQ-5D questionnaire to measure health-related quality of life for economic evaluation.^15^ In Big CACTUS an accessible (picture-based) version of the EQ-5D-5L questionnaire was administered to enable participants to understand the questions, and respond themselves^20,21^; this measure was informed by people with aphasia but is not yet psychometrically validated.

Responses to the accessible EQ-5D-5L questionnaire were combined with an algorithm developed by Van Hout et al.^22^ to calculate utility scores (a score describing health-related quality of life on a scale of 1 to −0.594, where a score of 1 represents perfect health and a score of 0 represents death). A utility score was assigned to the ‘Aphasia’ health state in the economic model, and utility increments (or decrements) were applied to the ‘Good response’ health states at six months, nine months and 12 months according to the difference in utility score change from baseline, between those in the ‘Good response’ state and those in the ‘Aphasia’ state. The utility increment associated with a good response at 12 months was extrapolated for the remainder of the modelled period. Utility scores were reduced over time to account for ageing.^23^ In each three-month cycle of the economic model, participants accrue QALYs according to the utility score of the health state that they reside in (e.g. spending three months in a health state with a utility score of 0.8 would accrue 0.2 QALYs (0.8 × 3/12)). QALYs were estimated in this way for the duration of the economic model, allowing total QALYs associated with each treatment strategy to be calculated. QALYs were discounted at an annual rate of 3.5%, according to NICE recommendations.^15^ Discounting is included in economic evaluation because benefits and costs that are incurred in the present are usually valued more highly than benefits and costs occurring in the future – discounting benefits reflects society’s preference for benefits to be experienced sooner rather than later.^15^

The EQ-5D-5L questionnaire is the latest version of the EQ-5D instrument, developed in 2011,^24^ which contains five questions about health-related quality of life, with five levels of response for each question. The EQ-5D-3L questionnaire was developed in the 1990s and contains five similar questions, with three levels of response for each question.^25^ The EQ-5D-5L has the advantage of an increased number of possible responses and so may be more sensitive, but there is currently disagreement about how to estimate utility scores (i.e. health-related quality of life scores) from it. A ‘tariff’ exists, allowing EQ-5D-5L questionnaire responses to be transformed into a utility score,^26^ but there are concerns about its validity.^22,27^ Therefore, NICE^27^ recommends using a mapping algorithm developed by Van Hout et al.^22^ to estimate utility scores using the EQ-5D-3L tariff from responses to the EQ-5D-5L questionnaire. We used the Van Hout et al.^22^ algorithm in our base-case analysis, but, recognising the disagreement around this, conducted pre-planned secondary analyses using alternative approaches. These included using the EQ-5D-5L tariff for England (developed by the Office for Health Economics (OHE)),^26^ and using an alternative mapping algorithm developed by Hernandez-Alava et al.^28^ which, like the Van Hout et al.^27^ algorithm, maps EQ-5D-5L questionnaire responses onto the EQ-5D-3L tariff to calculate utility scores.

Often economic analyses are conducted using proxy reports when participants are unable to complete standard health-related quality of life questionnaires. Hence, in an additional secondary analysis, we used utility scores calculated from standard EQ-5D-5L questionnaire responses completed on behalf of Big CACTUS participants by their informal carers.

### Resource use and costs

Costs included were consistent with the NHS and personal social services perspective taken. For computerised therapy, costs included computers and headsets (for those who needed these on loan from the NHS), StepByStep^©^ software, time spent by speech and language therapists and assistants delivering – and being trained to deliver – the intervention, and travel costs. Attention control costs included puzzle books and a staff member’s time spent phoning participants each month. Data on these were collected in Big CACTUS using activity logs^11^ and in our base-case analysis resource use estimates were based on these data. Because usual care was included in each of the intervention groups, we assumed that there would be no difference in these costs between treatment arms. Therefore, costs associated with usual care were not included in the economic evaluation.

Costs included were one-off and therefore were not extrapolated beyond the one-year trial period. In economic evaluation, costs that occur in the future are usually discounted to reflect society’s preference for costs to be incurred in the future rather than the present.^15^ However, when all costs are incurred in the first year of the analysis, costs are short-term and discounting is not required. The cost year was 2016/17. National unit costs were used to value resource uses^29^ (Table 1).

**Table 1.** Unit costs.

Item description	Unit cost (£)	References	Note
Laptop/tablet loan for six months (for participants without own computer)	69	Palmer et al.^11^	Unit cost calculated from average cost of a laptop/tablet purchased through the NHS (£690), divided by 10 users over shelf life.
StepbyStep^©^ software individual licence	250	Steps Consulting Ltd^30^	
StepbyStep^©^ software clinician licence	550	Steps Consulting Ltd^30^	
StepbyStep^©^ software clinician 5-licence bundle	2200	Steps Consulting Ltd^30^	
Headsets	14.50	Palmer et al.^11^	
Puzzle books	2.50	Palmer et al.^11^	
SLT band 7 cost/minute	0.90	Curtis and Burns^29^	Delivery of computerised therapy training
SLT band 6 cost/minute	0.75	Curtis and Burns^29^	Delivery of computerised therapy intervention
SLT band 5 cost/minute	0.57	Curtis and Burns^29^	Delivery of attention control intervention
SLTA band 3 cost/minute	0.41	Curtis and Burns^29^	Delivery of computerised therapy intervention
Travel cost/mile	0.45	Curtis and Burns^29^	

SLT: speech and language therapist; SLTA: speech and language therapy assistant; NHS: National Health Service.

We considered it possible that over time speech and language therapists and assistants would become more familiar with the software, and, consequently, would require less time to set-up and deliver computerised therapy. To represent this scenario, we ran a secondary analysis in which speech and language therapist and assistant costs were halved.

### Missing data

Data was missing for some of the clinical measures assessed in Big CACTUS (e.g. because some participants did not complete all questionnaires at some time-points). Where data are missing, it is common to impute what the missing values might have been, taking into account uncertainty around these imputations.^31,32^ Missing data for EQ-5D-5L utility scores and co-primary outcome scores was imputed with multiple imputation using a technique called predictive mean matching,^31,32^ described fully elsewhere.^11^

### Probabilistic analysis

Analyses were undertaken allowing for uncertainty in all of the model inputs (i.e. health state transition probabilities, utility (health-related quality of life) scores, resource use (cost) estimates) – that is, the analyses were undertaken ‘probabilistically’. For instance, our analysis of the response rates observed in Big CACTUS provides an estimate of the probability of achieving a ‘Good response’ for each treatment at each time point, but this estimate is not certain – thus requiring confidence intervals to be stated. Because the exact values of each parameter included in the economic model are unknown, probability distributions were placed around them and probabilistic analyses were used. This involved running the model thousands of times, each time randomly selecting a value from the distribution of each uncertain parameter in the model. Various different probability distributions exist – we used those typically used for model input parameters in economic modelling^33^: Normal distributions were assigned to utility score change parameters, beta distributions to transition probabilities and gamma distributions to resource use parameters. The model was run 10,000 times for the base-case analysis and for each subgroup and secondary analysis. Each model run provided an estimate of the costs and QALYs associated with each intervention and the average estimates of incremental costs and QALYs were used to provide the best estimate of the incremental cost per QALY gained (i.e. the ICER). We calculated 95% ‘credible intervals’ for incremental costs and QALYs, representing the interval within which the value of incremental costs and QALYs falls with a 95% probability. Cost-effectiveness planes and cost-effectiveness acceptability curves (CEACs) were used to graphically represent uncertainty, and demonstrate the probability that the competing interventions represent a cost-effective use of healthcare resources.

## Results

### Model input parameters and probabilistic analysis

Supplemental Table 1 presents input parameters used in the model. Computerised therapy plus usual care resulted in the highest proportion of good responses. The estimated utility score change (i.e. the health-related quality of life change) associated with a good response was negative at six months (–0.04, 95% CI: −0.09 to 0.01) and nine months (–0.02, 95% CI: −0.07 to 0.03), but was positive at 12 months (0.02, 95% CI: −0.03 to 0.07). There was 1% missing data for the accessible EQ-5D-5L questionnaire at baseline, and 18% at 12 months. There were no missing data for cost variables.

### Cost-effectiveness results

**Figure 2.**
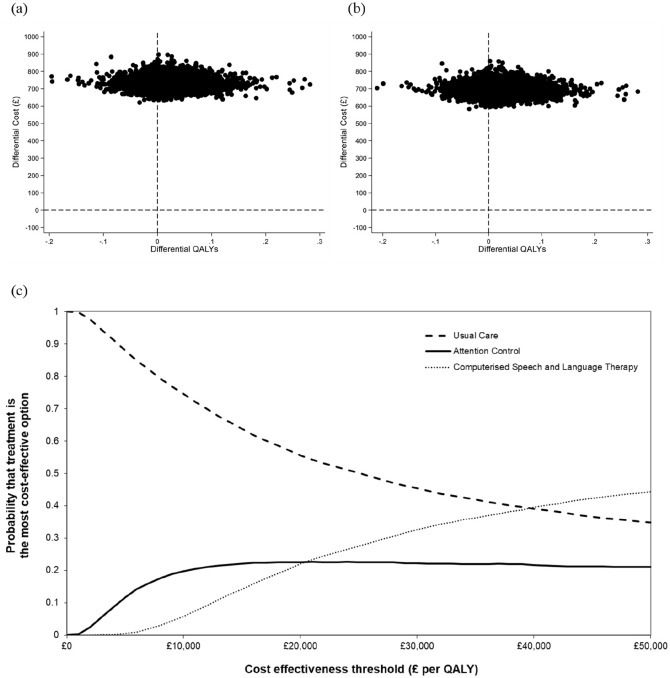
Cost-effectiveness planes for computerised therapy plus usual care compared to (a) usual care alone and (b) attention control plus usual care, and (c) cost-effectiveness acceptability curves (CEACs) – base-case analysis.

Table 2 presents results from the base-case analysis (representing our preferred assumptions for the full Big CACTUS population) and secondary analyses (representing alternative assumptions around key model inputs). In the base-case, the ICER for computerised therapy plus usual care versus usual care alone was £42,686 ($55,541) per QALY gained (incremental cost per-patient £732.73 ($953.39) (95% Credible Interval (CrI) £674.23–£798.05 ($877.27–$1,038.38)); incremental QALY gain per-patient 0.02 (95% CrI −0.05 to 0.10)). For computerised therapy plus usual care versus attention control plus usual care, the ICER was £40,164 ($52,259) per QALY gained. Attention control plus usual care was more expensive and produced fewer QALYs than usual care alone (in economic terms it was ‘dominated’). Figure 2 depicts cost-effectiveness planes and acceptability curves for the base-case analysis. Using a £30,000 ($39,035) per QALY gained threshold, the probability that computerised therapy plus usual care represents the most cost-effective treatment option was 32% (45% for usual care alone; 22% for attention control plus usual care).

**Table 2.** Cost-effectiveness results from base-case and secondary analyses – computerised therapy plus usual care compared to usual care alone, and compared to attention control plus usual care.

Analysis	Computerised therapy plus usual care cost (£)	Comparator cost (£)	Incremental cost [£]: Computerised therapy plus usual care versus comparator (95% credible interval)	Computerised therapy plus usual care QALYs	Comparator QALYs	Incremental QALYs: Computerised therapy plus usual care versus comparator (95% credible interval)	ICER (£ per QALY gained)
Comparator: usual care alone							
Base-case analysis	732.73	0.00	732.73 (674.23–798.05)	4.2164	4.1992	0.0172 (−0.05 to 0.10)	42,686
Using English EQ-5D-5L tariff	732.25	0.00	732.25 (673.19–797.84)	4.8537	4.8406	0.0132 (−0.04 to 0.09)	55,639
Using carer proxy EQ-5D-5L	733.06	0.00	733.06 (672.70–800.01)	3.5339	3.5084	0.0254 (−0.05 to 0.12)	28,819
Hernandez and Pudney^28^ EQ-5D mapping	732.96	0.00	732.96 (672.60–798.22)	4.1568	4.1358	0.0210 (−0.04 to 0.11)	34,921
SLT/SLTA costs halved	448.92	0.00	448.92 (411.50–495.12)	4.2164	4.1992	0.0172 (−0.05 to 0.10)	26,153
Comparator: attention control plus usual care							
Base-case analysis	732.73	38.14	694.59 (636.46–760.09)	4.2164	4.1991	0.0173 (−0.05 to 0.10)	40,164
Using English EQ-5D-5L tariff	732.25	38.17	694.09 (634.95–759.75)	4.8537	4.8402	0.0135 (−0.04 to 0.09)	51,308
Using carer proxy EQ-5D-5L	733.06	38.18	694.88 (634.58–761.87)	3.5339	3.5085	0.0254 (−0.05 to 0.12)	27,397
Hernandez and Pudney^28^ EQ-5D mapping	732.96	38.18	694.78 (634.94–760.21)	4.1568	4.1356	0.0211 (−0.04 to 0.11)	32,835
SLT/SLTA costs halved	448.92	38.19	410.78 (373.09–457.72)	4.2164	4.1991	0.0173 (−0.05 to 0.10)	23,753

QALY: quality adjusted life year; ICER: incremental cost effectiveness ratio; SLT: speech and language therapist; SLTA: speech and language therapy assistant.

Using different approaches to estimate utility scores resulted in markedly different ICERs, ranging from £28,819 ($37,498) to £55,639 ($72,395) per QALY gained for computerised therapy plus usual care compared to usual care alone (Table 2). Speech and language therapist time was the predominant cost driver – the 7.13 hours per-patient spent setting up and supporting computerised therapy contributed 44% of the total computerised therapy cost. Halving speech and language therapist and assistant costs reduced the ICER for computerised therapy plus usual care compared to usual care alone to £26,153 ($34,029) per QALY gained.

### Subgroup analysis

ICERs for participants with mild and moderate word finding difficulties at baseline were £22,371 ($29,108) and £21,262 ($27,783) per QALY gained respectively, for the comparison of computerised therapy plus usual care with usual care alone (Table 3). Computerised therapy plus usual care was more costly and less effective than usual care alone and attention control plus usual care for participants with severe word finding difficulty – in economic terms, in this subgroup computerised therapy plus usual care was dominated by usual care alone.

**Table 3.** Results of selected subgroup analyses.

Analysis	Subgroup	Computerised therapy plus usual care cost (£)	Comparator cost (£)	Incremental cost (£): Computerised therapy plus usual care versus Comparator (95% credible interval)	Computerised therapy plus usual care QALYs	Comparator QALYs	Incremental QALYs: Computerised therapy plus usual care versus Comparator (95% credible interval)	ICER (£ per QALY gained)
Parameters included in the economic								
Word finding difficulty (baseline)	Mild	653.49	0.00	653.49 (586.44–728.36)	4.2856	4.2564	0.0292 (−0.06 to 0.17)	22,371
	Moderate	814.44	0.00	814.44 (703.43–940.67)	4.3225	4.2842	0.0383 (−0.10 to 0.25)	21,262
	Severe	778.71	0.00	778.71 (674.77–890.79)	3.9971	4.0137	−0.0166 (−0.16 to 0.11)	Dominated
Comparator: attention control plus usual care								
Word finding difficulty (baseline)	Mild	653.49	40.17	613.32 (545.64–689.09)	4.2856	4.2658	0.0198 (−0.07 to 0.16)	30,911
	Moderate	814.44	37.78	776.65 (665.50–903.55)	4.3225	4.2657	0.0568 (−0.11 to 0.29)	13,673
	Severe	778.71	35.08	743.63 (640.27–856.15)	3.9971	4.0056	−0.0084 (−0.14 to 0.12)	Dominated

QALY: quality adjusted life year; ICER: incremental cost effectiveness ratio. In the full report of the Big CACTUS study,[11] ICERs for computerised therapy plus usual care in the moderate word finding difficulty subgroup were given as £28,898 per QALY compared to usual care alone, and £18,855 per QALY gained compared to attention control plus usual care. There was an error in these calculations and the figures given in this manuscript are corrected.

## Discussion

We demonstrate that, despite the (partial) clinical benefits observed in Big CACTUS, the cost-effectiveness of adding computerised therapy to usual care remains uncertain. Our incremental cost-effectiveness ratios are close to commonly accepted cost-effectiveness thresholds used in the United Kingdom. Fundamentally, it is unclear whether adding computerised therapy to usual care leads to a QALY gain compared to usual care alone. This is because the health-related quality of life benefit associated with a good response to computerised therapy was small and uncertain.

In Big CACTUS, adding computerised therapy to usual care led to a substantial, significant improvement in word finding ability compared to usual care alone and attention control plus usual care, but did not demonstrate improvements in functional communication.^11^ Improved word finding ability is represented in our economic model – more participants in the computerised therapy plus usual care group entered the ‘good response’ health state. However, there was no clear health-related quality of life gain associated with a good response, which caused the highly uncertain cost-effectiveness results. Given that computerised therapy was not found to improve functional communication in Big CACTUS, it is perhaps unsurprising that it did not result in clear improvements in health-related quality of life measured by the EQ-5D-5L questionnaire. A key challenge, and a priority area for further research, is to investigate ways in which improvements in word finding ability obtained through adding computerised therapy to usual care could be generalised into functional improvements, which might result in appreciable QALY gains.

It is notable that, as part of the computerised therapy intervention, it was intended that therapy assistants or volunteers would practise tasks to promote the use of new words in context. However, these tasks were only carried out for an average of 45 minutes per patient over the entire six month treatment period.^12^ This may have inhibited the generalisation of word finding improvements to functional conversation improvements, meaning that potential QALY gains were not realised.

Our analyses demonstrate that only very small QALY gains are required for adding computerised therapy to usual care to represent a cost-effective use of resources, because computerised therapy costs are low. The estimate of the QALY gain associated with computerised therapy was 0.02 in the analysis for the full Big CACTUS population, and was 0.03 and 0.04 in the mild and moderate word finding difficulty subgroups respectively (with substantial confidence intervals around these values). This difference was enough to change the interpretation of the cost-effectiveness results – the ICER was greater than NICE’s £30,000 ($39,035) per QALY gained threshold for the full Big CACTUS population, but was lower than that threshold in the mild and moderate word finding difficulty subgroups. Also, there is currently disagreement about how to calculate health-related quality of life scores (and therefore QALYs) from the EQ-5D-5L questionnaire^27^ and, in our analyses, using different approaches resulted in markedly different cost-effectiveness estimates. NICE^27^ currently recommends the Van Hout et al.^22^ algorithm for calculating utility scores from the EQ-5D-5L questionnaire, but other options are available.^26,28^ The different approaches result in small differences in health-related quality of life estimates, but in cases such as ours, where treatment costs are low, small changes in QALY estimates can lead to large changes in cost-effectiveness estimates.

The average cost of computerised therapy in Big CACTUS was £733 ($954) per participant. Speech and language therapists spent 7.13 hours on setup and support for each computerised therapy participant, and computerised therapy participants used the software for an average of 28 hours during the trial period.^11^ These costs are low: providing 28 hours of face-to-face speech and language therapy would cost approximately £1400 ($1822), almost twice as much as supporting an individual to practise independently with computerised therapy – although the relative effectiveness of face-to-face care is unknown.

There may be scope to further reduce the cost of computerised therapy, which could alter the conclusions of our cost-effectiveness analysis. If speech and language therapist and assistant costs could be halved, the ICER for adding computerised therapy to usual care compared to usual care alone would fall to £26,153 ($34,029) per QALY gained for the full Big CACTUS population. This may be possible – Big CACTUS participants were recruited between September 2014 and August 2016, when the StepByStep^©^ software was new and had teething issues and speech and language therapists were learning how to use it. Therapist support time and associated costs may be lower in an established clinical service. Whilst reducing therapist setup/support time could improve the cost-effectiveness of computerised therapy, their oversight is likely to remain important. Potentially speech and language therapists could assume a consultative role, guiding assistants and volunteers to personalise the software, and only adding more personally relevant words once a participant has demonstrated engagement with the intervention with an initial limited word set. This is in contrast to the use of qualified speech and language therapist time to personalise large word sets immediately upon initiation of the intervention, as was the case in Big CACTUS.

Whilst cost savings in the delivery of computerised therapy could be realised in reality, it is also possible that approaches for generalising word-finding benefits to functional conversation (and QALY) improvements might require a broader package of care – and therefore increased costs. However, given the low cost of computerised therapy, and the potential for increased benefits, such a package may represent a cost-effective use of healthcare resources.

The only previous evaluation of the cost-effectiveness of computerised therapy for post-stroke aphasia was undertaken alongside the CACTUS pilot study. This resulted in a much lower ICER for computerised therapy plus usual care compared to usual care alone (£3,127 ($4,069) per QALY gained).^10^ This is primarily because the utility gain associated with a good response was estimated to be much higher using pilot study data (0.07 (CI −0.15 to 0.29), compared to 0.02 (CI −0.03 to 0.07) using Big CACTUS data). Confidence intervals around this estimate have been reduced by Big CACTUS, but centre around the lower end of the interval estimated in the pilot study. Consequently, the ICER estimated using Big CACTUS data is much higher than that estimated using pilot study data, and cost-effectiveness estimates remain uncertain.

Our economic evaluation adhered to good practise guidelines and was based on a well-conducted full-scale RCT to enable a robust assessment of the cost-effectiveness of adding computerised therapy to usual care. A particular strength of the Big CACTUS study was the chronicity of the study participants – median time since stroke was approximately two years, and therefore the computerised therapy intervention was truly tested on people who were experiencing aphasia long after stroke. In this context, the clinical results observed are particularly encouraging.

Using an accessible version of the EQ-5D-5L questionnaire represents both a strength and a weakness of this study. It addressed concerns around the validity of the standard EQ-5D-5L questionnaire for people with aphasia^20,21^ and allowed health-related quality of life data to be collected directly from patients, avoiding well-known issues associated with collecting such information by proxy.^34^ However, further research should also assess whether the accessible version of the EQ-5D-5L questionnaire is a valid and responsive tool for measuring health-related quality of life in people with aphasia. The EQ-5D questionnaire was used in Big CACTUS and in our assessment of cost-effectiveness because it is preferred by NICE.^15^ As opposed to disease-specific measures, generic, preference-based measures such as the EQ-5D questionnaire provide a basis for making consistent resource allocation decisions throughout health systems, but it might be argued that the EQ-5D questionnaire does not well represent the quality of life constructs that might be expected to change through improved communication. NICE recognises that in some cases the EQ-5D questionnaire may not be appropriate, but requires qualitative and empirical evidence to support such an argument.^15^ In addition, given that computerised therapy plus usual care did not lead to an improvement in functional communication as measured using Therapy Outcomes Measures in Big CACTUS, it is questionable whether any quality of life measure would have shown an improvement. Finally, self-managed therapy allows people to exercise choice over their own health care – it is unclear whether this empowerment would be captured by disease-specific outcome measures, or by generic questionnaires such as the EQ-5D.

A limitation of our analysis is that only direct intervention costs associated with computerised therapy and attention control were included, implying an assumption of equal costs associated with usual care across the intervention groups. This assumption was made because the computerised therapy investigated in Big CACTUS was intended as an addition to usual care, rather than a replacement for it, and usual care was maintained in each intervention group. However, we recognise that receiving computerised therapy could have an impact on other care received by people with aphasia. The CACTUS pilot study collected information on a wide range of resource use (such as medication, primary care and hospital care) but did not show important differences between treatment groups,^10^ and for this reason such information was not collected in Big CACTUS. However, information on usual speech and language therapy care received during Big CACTUS *was* collected, allowing an assessment of this aspect of usual care between randomised groups. The amount of care received was low, reduced through the trial period, and was comparable between groups.^11^ However, there was an indication that slightly less usual speech and language therapy was received in the computerised therapy plus usual care group, compared to usual care alone (mean 3.2 hours across the six-month intervention period in the computerised therapy plus usual care group, compared to 3.8 hours in the usual care alone group).^11^ For context, a one-hour reduction in speech and language therapist-provided usual care equates to a cost saving of approximately £50 ($65) per patient, which would slightly reduce the ICER for computerised therapy plus usual care compared to usual care alone, from £40,164 ($52,259) per QALY gained to approximately £40,000 ($52,046) per QALY gained. Therefore, we expect that the impact of including usual care costs in our economic evaluation would have been minimal.

Including only direct intervention costs in our analysis also implies an assumption that there are no differences in indirect resource use associated with the intervention groups. Hence, potential knock-on effects on other healthcare appointments were excluded. We made this decision because Big CACTUS did not collect data on wider resource use, due to the pilot study finding no important differences in indirect resource use associated with computerised therapy compared to usual care.^10^ This is in line with our expectation that the computerised therapy intervention evaluated would not have knock-on effects on other healthcare resource use.

It is important to note the potentially limited generalisability of our analysis. The computerised therapy studied focussed only on the treatment of word finding with one piece of software, and required speech and language therapists to tailor and personalise the software and train and support volunteers and assistants to support patients. Different approaches to using computer therapy may have a different cost-effectiveness profile.

## Conclusion

Although adding computerised therapy to usual care improves personally relevant word finding compared to usual care alone this does not translate into appreciable health-related quality of life gains – with estimated gains small and uncertain. Consequently, the cost-effectiveness of the intervention is uncertain. Further research is required to investigate how word finding improvements might lead to quality of life gains.

**Clinical messages**

- Computerised therapy is unlikely to be cost-effective for the general population of people with aphasia post stroke. It is more likely to be cost-effective for people with mild or moderate aphasia.
- Computerised therapy improves personally relevant word finding but this does not translate into appreciable health-related quality of life gains.

## Supplemental Material

sj-pdf-1-cre-10.1177_0269215520975348 – Supplemental material for Self-managed, computerised word finding therapy as an add-on to usual care for chronic aphasia post-stroke: An economic evaluation Click here for additional data file. Supplemental material, sj-pdf-1-cre-10.1177_0269215520975348 for Self-managed, computerised word finding therapy as an add-on to usual care for chronic aphasia post-stroke: An economic evaluation by Nicholas R Latimer, Arjun Bhadhuri, Abu O Alshreef, Rebecca Palmer, Elizabeth Cross, Munya Dimairo, Steven Julious, Cindy Cooper, Pam Enderby, Marian C Brady, Audrey Bowen, Ellen Bradley and Madeleine Harrison in Clinical Rehabilitation
