# Supplementary material for: Self-managed, computerised word finding therapy as an add-on to usual care for chronic aphasia post-stroke: An economic evaluation
Source: Clin Rehabil. 2020 Nov 24;35(5):703–17. doi: 10.1177/0269215520975348 (PMC8073872; doi:10.1177/0269215520975348)
Supplement: sj-pdf-1-cre-10.1177_0269215520975348 – Supplemental material for Self-managed, computerised word finding therapy as an add-on to usual care for chronic aphasia post-stroke: An economic evaluation [file sj-pdf-1-cre-10.1177_0269215520975348.pdf]

Supplemental Table 1: Parameters included in the economic model and their values in the base-case cost-effectiveness analysis

Parameter	Mean	Distribution (parameters)	95% CI	Source	Mean Cost (£)*
**Computerised therapy resource use parameters**					
Proportion who need a computer	0.68	Beta(66,31)	0.59-0.77	[11]	46.95
Proportion who need a headset	0.33	Beta(32,65)	0.24-0.43	[11]	4.78
SLT time: setup/supporting computerised therapy (hours)	7.13	Gamma(0.54)	6.06-8.19	[11]	320.72
SLT time: supervising/training SLTs/volunteers (hours)	1.83	Gamma(0.14)	1.55-2.10	[11]	82.47
SLTA time: with computerised therapy participants (hours)	2.73	Gamma(0.28)	2.17-3.28	[11]	68.24
SLTA time: under supervision of SLT (hours)	1.11	Gamma(0.12)	0.86-1.35	[11]	27.79
SLT/SLTA travel (mileage)	127.52	Gamma(13.50)	100.67-154.35	[11]	57.38
Utilisation rate of software licenses	0.50	Beta(5,5)	0.21-0.79	Assumption	109.84
Number of patients who benefit annually from training 27 SLTs to deliver computerised therapy	55.43	Gamma(11.09)	35.99-79.58	[11] Standard error equal to 20% of the mean	10.33
**Attention control resource use parameters**					
Puzzle books	4.35	Gamma(0.20)	3.96-4.74	[11]	10.89
SLT time: administering attention control (hours)	0.80	Gamma(0.05)	0.70-0.89	[11]	27.29
**Computerised therapy transition probabilities**					
Probability good response (0-6 months)	0.78	Beta(73,21)	0.67-0.85	[11]	
Probability relapse (6-9 months)	0.19	Beta(14,59)	0.11-0.30	[11]	

Probability new good response (6-9 months)	0.33	Beta(7,14)	0.15-0.57	[11]
Probability relapse (9-12 months)	0.08	Beta(5,61)	0.03-0.17	[11]
Probability new good response (9-12 months)	0.39	Beta(11,17)	0.22-0.59	[11]
Probability relapse (12 months onwards)	0.08	Beta(5,61)	0.03-0.17	[11]
**Usual care transition probabilities**				
Probability good response (0-6 months)	0.46	Beta(45,52)	0.36-0.56	[11]
Probability relapse (6-9 months)	0.22	Beta(10,35)	0.12-0.37	[11]
Probability new good response (6-9 months)	0.37	Beta(19,33)	0.24-0.51	[11]
Probability relapse (9-12 months)	0.22	Beta(12,42)	0.12-0.35	[11]
Probability new good response (9-12 months)	0.37	Beta(16,27)	0.23-0.53	[11]
Probability relapse (12 months onwards)	0.22	Beta(12,42)	0.12-0.35	[11]
**Attention control transition probabilities**				
Probability good response (0-6 months)	0.49	Beta(49,40)	0.38-0.60	[11]
Probability relapse (6-9 months)	0.28	Beta(11,28)	0.15-0.44	[11]
Probability new good response (6-9 months)	0.35	Beta(14,26)	0.21-0.51	[11]
Probability relapse (9-12 months)	0.19	Beta(8,34)	0.09-0.34	[11]
Probability new good response (9-12 months)	0.19	Beta(7,30)	0.08-0.36	[11]
Probability relapse (12 months onwards)	0.19	Beta(8,34)	0.09-0.34	[11]
**Utility scores (accessible EQ-5D-5L)**				
Utility score (Aphasia health state)	0.61	Beta(650.36, 407.99)	0.59-0.64	[11]

Difference in utility associated with good response	At 6 months	-0.04	Normal(0.03)	-0.09-0.01	[11]
	At 9 months	-0.02	Normal(0.03)	-0.07-0.03	[11]
	At 12 months	0.02	Normal(0.03)	-0.03-0.07	[11]
**Probability of death**					
Annual, for all treatment groups		0.10	Beta(233,2203)	0.09-0.11	[18]

Note: parameters for Beta distribution – alpha, beta; parameter for Gamma distribution – standard error; CI – confidence interval. SLT - Speech and Language Therapist; SLTA - Speech and Language Therapy Assistant
*Mean costs presented in this table represent the mean cost of each resource use parameter included in the economic model, based upon the mean resource use multiplied by its unit cost (given in Table 1). The mean cost for software licenses requires a more complex calculation that is not presented here, as does the mean cost per patient of training SLTs to provide the intervention. In addition, these costs are deterministic – that is, they simply represent the unit cost multiplied by the mean resource use value, and do not take into account uncertainty. Uncertainty is taken into account in the economic model results presented in Tables 2 and 3, through probabilistic analysis. Therefore, whilst the total intervention costs for the computerised therapy and the attention control approximately equal the sums of the mean costs included in this table, they do not exactly match those presented in Table 2.
